# Comparative *In Vivo* Effects of Hemoglobin-Based Oxygen Carriers (HBOC) with Varying Prooxidant and Physiological Reactivity

**DOI:** 10.1371/journal.pone.0153909

**Published:** 2016-04-20

**Authors:** Vlad Al. Toma, Anca D. Farcaș, Ioana Roman, Bogdan Sevastre, Denisa Hathazi, Florina Scurtu, Grigore Damian, Radu Silaghi-Dumitrescu

**Affiliations:** 1 Biological Research Institute, Cluj Napoca, 400113, Romania; 2 Department of Chemistry and Chemical Engineering, Babes-Bolyai University, Cluj-Napoca, 400028, Romania; 3 National Institute for Research and Development of Isotopic and Molecular Technologies, 400293, Cluj-Napoca, Romania; 4 Department of Pathophysiology, University of Agricultural Sciences and Veterinary Medicine, Cluj Napoca, 400372, Romania; 5 Department of Physics, Babes-Bolyai University, Cluj-Napoca, 400028, Romania; Oklahoma State University, UNITED STATES

## Abstract

A series of hemoglobin-based oxygen carrier candidates (HBOC), previously noted for their differences in prooxidative and physiological reactivity, were compared in terms of the negative effects displayed upon injection in Wistar rats. At the concentrations tested, antioxidant strategies based on albumin as well as based on rubrerythrin appear to offer observable physiological advantages.

## Introduction

Artificial oxygen carriers (typically hemoglobin-based, HBOCs), are generally based on (bio)chemical modifications of hemoglobin (Hb) purified from outdated human blood or from bovine blood[1–3]. Such modifications include intramolecular crosslinking aimed to avoid tetramer dissociation (e.g., with acetaldehyde, bis(3,5-dibromosalicyl) fumarate- DBBF [4]), intermolecular crosslinking with bifunctional agents (e.g., glutaraldehyde, glycolaldehyde, oATP, disuccinimidyl suberate DSS[5–8]), and large-molecular-weight polymers (polyethylene glycols PEG, starch, and others[4, 5, 7, 9]). Additionally, in a different line of study, fluorocarbon-based oxygen carriers are also explored[3, 9].

The HBOC side-effects include, among others, NO depletion (via reaction with oxy Hb, leading to nonfunctional and toxic metHb) and a closely-linked hypertension[1, 10]. Attempts to limit this reaction have relied either on preventing extravasation of Hb across the endothelial vascular wall (increasing molecular size of the HBOC), or on genetically engineering the Hb in order to slow down its reaction with NO[4, 11, 12]. Besides NO, the deoxy and especially the met forms of Hb also interact with reactive oxygen species (ROS) such as superoxide (O_2_^•-^) or hydrogen peroxide (H_2_O_2_). These processes yield ferryl (Fe^IV^) Hb[11], a highly oxidizing species which not only degrades the Hb peptide and heme, but also amplifies the stress response by attacking other biomolecules and accidentally generating others (e.g., prostaglandins).

In this context, Hb cross-linked with glutaraldehyde (HbGL) has served as primer for producing a complex with endogenous enzymes (superoxide dismutase–catalase–carbonic anhydrase), as a strategy for limiting the oxidative and nitrosative stress produced by HbGL [13, 14] as well as for ensuring an efficient acid-base balance[15–17]. Another approach along this same line is HbGL conjugated with fibrinogen, which affords an oxygen carrier with platelet-like function, effective *in vivo* with rats[18].

Bovine HbGL preparations were tested extensively for applications as blood substitutes (e.g. Biopure, OPK Biotech), including clinical trials (phase III)[19, 20], and were approved for limited human use in South Africa (HemoPure)[4, 21]. Nevertheless, to our knowledge HbGL is not currently in use,—possibly due to its adverse effects such as oxidative stress, acidosis, endothelial cells disorders, cerebral ischemia, myocardial infarction and increased morbidity. Polyethylene glycol surface-conjugated human hemoglobin (PEG-Hb) clinical trials have gone as far as phase III[22, 23]. Submicron hemoglobin lipid vesicles have also been submitted to clinical trials. An advantage of these vesicles would be the fact that by efficiently packing the Hb they require lower volumes to be injected in the body, compared to the simpler non-encapsulated HBOCs[24].

Beyond instructive insight into the biochemistry and into the *in vitro* behaviour of HBOCs and of their components[25], the still-extant clinical side-effects are an argument for more detailed exploration of HBOC performance in animal models–in the hope of paving the way for better-informed choices in the design of better HBOC candidates[3, 26–34]. We have previously reported upon the preparation of a range of HBOCs based on bovine Hb with wide differences in prooxidant reactivities[5, 7, 25, 35–38], and on their effects on human cell cultures (human umbilical vein endothelial cells, HUVEC, and to some extent lymphocytes)[5, 25, 36–38]. All of these preparations have large molecular weights–the main differences consisting in the autooxidation rates and in the varying abilities to resist damage inflicted by hydrogen peroxide. Such a library of HBOC candidates would make an ideal candidate for testing the hypothesis that prooxidant reactivity is a key factor controlling the negative side-effects in blood substitutes. The objective of the present study is indeed this verification, by testing a library of HBOCs of various prooxidant reactivities. We have previously demonstrated that there is a general tendency of chemically-derivatized Hb’s (with GL, oATP, periodate-oxidized starch, oxidized PEG, or disuccinimidil suberate) to manifest *in vitro* pro-oxidant properties as illustrated by the autooxidation rates and by the peroxide reactivity of the ferric form (including the quantity of the free radical generated in such processes); nevertheless, co-polymerization of hemoglobin with serum albumin alleviates this problem completely.[35, 36] Likewise, copolymerization with Rbr was shown to improve the resistance against hydrogen peroxide in glutaraldehyde-polymerized Hb. According to these lines of thought, one would expect to also identify direct and indirect symptoms pointing to an advantage of Hb-BSA or Hb-Rbr copolymers over HbGL or HboATP in *in vivo* experiments. The present study thus aims to test this working hypothesis. A variety of parameters are monitored, many of which may suffer indirectly from the HBOC redox reactivity, via such mechanisms as imbalances in signaling molecules fortuitously produced in excess or depleted by the Hb heme (e.g., prostaglandins, NO, hydrogen peroxide, superoxide). Also, the HBOC iron may act as a form of stress with effects ranging as far as liver, spleen, and others. Such imbalances may cause discrete physiological effects at the level of distinct organs as well as generally throughout the cardiovascular system. The present manuscript thus attempts to build towards an understanding of the extent to which such changes will control the experimentally-measurable outcomes of HBOC use *in vivo*.

## Materials and Methods

### Chemicals

The reagents included in standard assay packets with colorimetric and kinetic methods were obtained from BioMaxima S.A., Lublin, Poland. Thiobarbituric acid and malondialdehyde standards were purchased from the Sigma-Aldrich Company, St. Louis, MO, USA. Test cards for the Epoc analyzer were obtained from Epocal Inc., Ottawa, Canada. Ketamin was obtained from CP-Pharma, Burgdorf, Germany; xylazine was obtained from Bioveta a.s, IvanovicenaHané, Czech Republic. Neutral formalin solutions were purchased from Chemical Company S.A., Iasi, Romania. All other chemicals and solvents used in the study were of analytical grade.

### Blood substitutes

Bovine hemoglobin,[39] recombinant *Desulfovibrio vulgaris* rubrerythrin (Rbr)[40] and *Clostridium acetobutylicum* NADH/rubredoxin oxidoreductase (NROR) [40] were purified as previously described. Proteins were manipulated in 137 mM NaCl, 2.7 mM KCl, 12 mM NaH_2_PO_4_, pH 7.4 (phosphate buffer saline, PBS); their concentrations are given per monomer (i.e., per active site). Bovine serum albumin (BSA fraction V, from Sigma, Germany) was used as provided without further purification.

The UV-vis spectra were recorded on Cary 50 (Varian, Inc.) instruments.

Hb was polymerized with glutaraldehyde (GL) (Sigma-Aldrich) following previously described protocols[7, 34, 37]. OxyHb (1mM) in PBS was mixed with 8 mM GL and allowed to react for 2 h at 4°C under mechanical stirring. For copolymerization, BSA or Rbr/NROR was added to the reaction mixture prior to GL, at molar ratios of 2:1 (BSA/Hb), 1:30 (Rbr/Hb), and 1:1:30 (Rbr/NROR/Hb), respectively. A freshly-prepared solution of borohydride was then added (at a 2-fold molar excess with respect to the initial GL concentration) in order to reduce the Schiff bases and the excess of GL. The product was dialyzed in 50 mM Tris buffer with 150 mM NaCl, pH 7.4, in order to remove excess NaBH_4_, the unreacted cross-linker, and side-products of the reduction (borates and related compounds). For derivatization with oATP, previously described protocols were likewise followed[5, 35].

### Experimental animals

The experiments and animals welfare were conducted according to the *Guide for the Care and Use of Laboratory Animals* (Department of Health Education, and Welfare, National Institute of Health, 1996), and followed the guidelines of the European Communities Council Directive (2010/63/UE Directive). The animal testing and experiments were conducted according to the approval of the Ethics Committee and Animal Protection for experiments from the Institute of Biological Research, NIRDBS branch, Cluj-Napoca, Romania. (Decision 1/28.02.2013). Healthy adult male white Wistar rats weighing 180±30 g, age 24 weeks, F1 generation, were purchased from the zoobase of the Iuliu Hatieganu University of Medicine and Pharmacy in Cluj-Napoca, and maintained in the zoobases of the Biological Research Institute and of the Molecular Biology and Biotechnology Department, Faculty of Biology and Geology, Babes-Bolyai University, Cluj-Napoca.

### Study design

The experiment was performed on 7 groups of male Wistar rats, differentiated as follows by the administered HBOC: native hemoglobin (nHb), polymerized hemoglobin (HbGL), copolymerized hemoglobin with BSA (HbBSA), copolymerized hemoglobin with rubrerythrin (HbRbr), copolymerized hemoglobin with rubrerythrin and NROR (HbRbrNROR) and polymerized hemoglobin with oATP (HboATP), plus control group (control) (untreated rats). Each group consisted of 10 animals, of the same gender and as similar as possible in terms of weight. The animals were purchased just before the experiment and were housed in high quality laboratory cages designed for small rodents, (polypropylene and a grill of stainless steel, size 290/178/160 mm L/W/H supplied by Lignifer, Hungary), with no more than 5 rats/cage. The bedding material consisted of wood chips. The animals were housed in hygienic conditions, under a 12/12 h light/dark cycle, at 20°C, with no noise. Handling was performed quietly and gently/slowly, always by two persons (VAT and ADF, supervised by IR). The standard diet (granulated fodder for rodents, from Cantacuzino Institute, Bucharest, Romania) and tap water were *ad libitum*; every cage had its own water bottle and food containers. The experimental treatments were carried out in a different room than the one employed for housing the animals.

### Experimental procedures

HBOCs were administered *via* intravenous injection (0.2 mL/ animal) á jeun under anesthesia (ketamine-xylazine, see also below). The experiment lasted for two days from this point. There animals were monitored every 10 minutes for the first two hours after administration of the HBOC, and then every two hours for the next 8 hours, and then again at 24 hours; semiologically, no change was noted in the animals throughout this time, and no unintended deaths occurred at any point during the experiment. At the end, the animals were anesthetized with a ketamine-xylazine cocktail (60 mg ketamine and 7.5 mg xylazine respectively/ kg b.w.) to ensure a neuroleptanalgesia which keeps the animal in proper condition for ~30 minutes so that venous blood may be conveniently collected from the orbital plexus in heparinized syringes or in EDTA anticoagulant tubes (50μL EDTA 4% for 500 μL fresh blood). Heparinized samples were processed using a multiparameter biochemistry analyser (Epoc Reader) similar to the ASTRUP microequipment. This technology is based on electrochemical assay signals and quality control signals which are read by detectors in the Epoc Reader and allow the investigation of the acid-base status of the body (pH, pO_2_, pCO_2_, cHCO_3_, BE(ecf), BE(b)), as well as glucose and ions such as Na^+^, K^+^ and Ca^2+^. Blood samples collected in EDTA anticoagulant tubes were labeled and immediately submitted for hematological analysis. Complete Blood Counts, including white blood cell count (WBC), the number and percentage of granulocytes (GRA), middle cells (MID), lymphocytes (LYM), red blood cells count (RBC), hemoglobin concentration (HGB), hematocrit (HCT), mean corpuscular hemoglobin (MCH), mean corpuscular volume (MCV), mean corpuscular hemoglobin concentration (MCHC), red blood cell distribution width (RDW), platelet count (PLT), thrombocytocrit (PCT), medium platelet volume (MPV), and platelet distribution width (PDW), were performed using an Abacus Junior Vet automatic analyzer (Diatron Messtechnik, Budapest, Hungary).

After the above-described initial blood collection, the animals, still under anesthesia, were euthanized by decapitation in order to perform the second blood collection, this time from the jugular vein. The serum from this blood was separated by centrifugation and analyzed for total protein content, transferrin, catalase, concentration of thiobarbituric acid reactive substances (TBARS), creatinine, urea, uric acid, iron and phosphate, using a semi-automatic biochemistry analyzer (Evolution 2000).

For iron histochemical analyses, the organs (liver, spleen and kidney) were collected by animal dissection, and fixed in neutral formalin solution (10%) for 42 hours. Then, the organs were embedded in paraffin and sectioned at 5 μm with a microtome (Reichert Austria). The sections were histoprocessed in a series of alcohol and xylene baths, and then stained by Pearl’s method. The microscopy images were obtained with a Carl-Zeiss research microscope, using a CCD photocamera (MDC200). The image magnification was 400x. Iron complexes are stained in nuances ranging from red to black.

EPR measurements were performed on venous blood samples collected from the rats at 10 minutes after intravenous injection with HBOC. The blood was collected into sterile tubes that contained 20 μL of citrate (to attain a concentration of 2% citrate in 500 μL collected blood) and immediately transferred into EPR tubes and frozen in liquid nitrogen. A Bruker EMX Micro spectrometer with a liquid nitrogen cooling system was employed. Instrument conditions: microwave frequency, 9.43 GHz, microwave power, 15.89 Mw; modulation frequency 100 kHz, modulation amplitude, 3G; sweep rate 22.6 G/s, time constant 81.92 ms, average of two sweeps for each spectrum, temperature 100K.

### Statistical methods

All data are reported as the mean ± SEM. The Gaussian distribution was checked by the Shapiro-Wilk normality test. One-way analyses of variance ANOVA were performed, followed by post hoc Dunnett’s range test procedures. Statistical significances were at p<0.05 (95% confidence interval). Statistical values were obtained using GraphPad Prism version 5.0 for Windows (GraphPad Software, San Diego California USA).

## Results

An amount of 0.2 mL of HBOC was injected per animal. This constitutes ~ 2% of the total blood volume of the rat, under conditions where the average weight of the set of animals in the present series of experiments is 180 grams, of which the blood represents 7%. This ratio of HBOC is ten times lower than the (minimal) 20% that would be used in order to treat a hemorrhage case. As such, the experiments reported herein serve an exploratory purpose in terms of identifying potential systemic inflammation problems, or potential major metabolic imbalances associated with newly-developed HBOC containing relatively exotic proteins such as rubrerythrin, NROR, or even BSA, for which at the onset one needs to assess for any strong physiological response. This approach avoids for the moment the requirements of using (1) much larger amounts of HBOC and (2) the technically and ethically more complex procedure of hemorrhage prior to injection (hemorrhage would be a pre-requisite when attempting to inject HBOC to 20% of the total blood volume, given the internal volume limitations of the organism’s cardiovascular system).

Complete Blood Counts (cf. Table 1) reveal variations among experimental groups which, while generally statistically significant, remain mainly confined within the normal range for the most of the parameters.

**Table 1:** Hematological parameters of Control and experimental groups. Values are expressed as mean ± SEM.

Parameters	Control	nHb	HbGL	HbBSA	HbRbr	HbRbrNROR	HboATP
LYM (10^9^/L)	4.8±0.1	5.5 ±0.5	4.9 ±0.3	3.9 ± 0.1*#	4.4 ± 0.1*#	4.5 ± 0.1*#	4.4 ±0.3
WBC (10^9^/L)	6.6 ±0.1	5.9 ±0.4	6.0±0.3	5.1±0.1*#	6.9±0.3	6.6 ± 0.1 #	6.2± 0.1*
GRA (10^9^/L)	1.4 ±0.02	1.6 ± 0.1*#	1.5±0.1	1.6 ± 0.1#	1.5±0.12#	1.7 ±0.04*#	1.1±0.01*#
GRA %	18.1 ±0.5	18.8± 0.9#	20.6± 0.7*	19.8 ± 0.3*#	20.0± 0.9#	25.3± 0.2*#	21.9± 0.5*#
RBC (10^12^/L)	8.7 ±0.04	8.9 ± 0.1*#	9.7± 0.1*	9.6± 0.1*	9.7 ± 0.1*	9.9 ±0.02*	9.1 ±0.1*#
HGB (g/L)	154.5±1.7	162.8± 1.9*	167.1 ± 1.2*	168.6± 0.4*	169.0± 1.3*	169.6 ± 1.0*	159.2± 0.8*
HCT (%)	47.0 ±0.3	50.7 ± 0.5*#	51.0± 0.3*	51.7 ± 0.4*	50.7± 0.4*	50.6± 0.3*	49.8± 1.1*#
MCV (fL)	54.0±0.31	55.2± 0.8#	52.2± 0.7*	51.7±0.48*	54.0±0.4	53.0± 0.5*	52.2± 0.7*
MCH (pg)	18.1±0.1	18.2± 0.2#	17.4± 0.2*	17.1± 0.2*	17.0 ± 0.2*	17.0±0.14*	17.6± 0.1*
PLT (10^9^/L)	542.2±17.0	1070.7 ± 11.0*#	1033.0± 42.7*	1000.3 ± 7.8*	1025.7 ± 5.2*#	895.3± 18.0*	1007.7 ± 2.3*#
PCT (%)	0.4 ±0.02	0.7±0.01*#	0.7 ±0.03*	0.7 ±0.02*	0.8 ±0.01*#	0.7 ±0.02*	0.8 ±0.01*#

* Significant at *p* < 0.05 as compared to Control

# Significant at *p* < 0.05 as compared to HbGL

The smallest (2–5%) increases in RBCs were in the nHb (8.88±0.08 10^12^/l) and HboATP (9.08±0.13 10^12^/l) (p<0.05) groups—incidentally the ones displaying the highest *in vitro* prooxidant reactivity. In the other groups (HbGL, HbBSA, HbRbr, HbRbrNROR) the values were ~10–15% larger than in the control group (highest in HbRbrNROR, 9.87±0.02 10^12^/l p<0.001), all of which may, however, be judged to be within normal ranges.

HGB expectedly followed similar trends to RBC: it was slightly (3–5%) elevated in groups nHb (162.80±1.94 g/L)(p<0.01) and HboATP (159.25±0.75 g/L) (p<0.05), and further higher (8–10%) in the other groups (HbGL, HbBSA, HbRbr, HbRbrNROR); HbRbrNROR, once again, revealed the highest value (169.58±0.97 g/L)(p<0.001).

HCT was elevated by 6–10% in all experimental groups compared to control; the values were nevertheless very similar to each other, and situated at the higher level of the normal limit. They showed a tight variation between 49.80±1.09 (p<0.01) in HboATP and 51.67±0.48 (p<0.01) in HbBSA.

The red blood cell indices, MCV and MCH, showed inconstant variations among experimental groups, but were within the reference range of the species (variations vs. control of 2–4% and 1–6% respectively, for the two parameters).

WBC counts remained in the normal range, and the variations were insignificant for most of the animal groups, with the exception of HbBSA which had lower values of 5.11±0.05 (10^9^/l) (p<0.01)–a 20% decrease compared to the control group. The lowest change (1%) was in the HbRbrNROR group. LYM followed the same trend as the WBCs; HbBSA showed the smallest value (-20% compared to control, at 3.87±0.08) (p<0.001). This similarity in trends between WBC and LYM is not surprising, as long as the lymphocytes are the predominant white blood cell population in rodents[41]. GRA, the category including the granular leukocytes largely dominated by neutrophils, was elevated by up to ~10–20% in almost all groups, except for the HboATP which had a ~20% lower concentration of 1.12±0.01 10^9^/l (p<0.001). The highest concentration was found in HbRbrNROR (1.69±0.04 10^9^/l) (p<0.01).

PLTs were elevated in all experimental groups, almost twofold increased compared to the control; the highest value were in the nHb group (1070.67±11.02) (p<0.001), and the lowest (65% increase over control) in HbRbrNROR.

PCT was increased significantly (by 60–80%) in all groups.

Before the experiment, IDR (Intra Dermic Reaction) tests were performed with all 6 HBOCs, on 3 animals each. Since no adverse reaction was noted, the intravenous experiments were carried out with the following results. Immunological parameters (IgA, IgG, IgM and CRP, cf. Table 2) showed statistically significant increases in the experimental groups, in tandem with clotting parameters (fibrinogen and PT). Nevertheless, the newer variants (copolymerization with albumin—HbBSA, or with the protective redox system Rbr/NROR—HbRbrNROR) show no notable changes in immunoglobulins compared to HbGL.

**Table 2:** Immunological and clotting parameters of Control and experimental groups. Values are expressed as mean ± SD.

Param.	Control	nHb	HbGL	HbBSA	HbRbr	HbRbrNROR	HboATP
IgA (mg/dL)	1.0 ± 0.2	6.6 ±0.1*	8.5 ±0.5*	6.3±0.8*	7.3 ±0.3*	6.0 ±1.1*	8.2 ±0.2*
IgG (mg/dL)	70.0 ± 4.3	90.8 ± 2.33*#	206.6 ± 3.71*	162.0 ±13.1*#	277.1 ± 7.0*#	230.1 ± 9.5*#	202.5 ± 8.6*
IgM (mg/dL)	19.0±1.2	64.3 ±3.0*	57.0 ±4.0*	47.0 ±5.72*	30.2 ±0.5*#	54.0 ± 3.2*	60.6 ± 0.8*
C3C (mg/dL)	54.5 ±2.8	54.6 ±16.5	55.50±4.9	29.75±3.1*#	37.5 ±5.9^a^*	66.4 ±1.1*#	70.6 ±10.3
CRP (mg/dL)	41.5 ±1.8	242.3 ±10.3*	254.8 ±18.3*	197.2 ±8.8*#	218.2 ±15.1*	333.4 ±20.7*	78.0 ±3.7*#
Fibrinogen (mg/dL)	80.6±3.1	213.2± 6.5*#	172.0±3.1*	298.4 ± 11.5*#	155.6±2.4*#	304.0±7.4*#	261.2 ± 6.7*#
PT (s)	10.6±0.2	10.0±0.1*	9.9±0.1*	10.4±0.2#	10.6±0.1#	10.0±0.1*	10.3±0.1#
aPTT (s)	16.9±0.5	16.8±0.4	16.0±0.2	15.9±0.6	15.7±0.1#	15.8±0.1	16.4±0.3

* Significant at *p* < 0.05 as compared to Control

# Significant at *p* < 0.05 as compared to HbGL

The aPTT levels remained near normal, with no statistically significant changes (Table 2).

C3C levels revealed significant variations between experimental groups, decreasing in HbBSA (29.75±3.17) (p<0.001), HbRbr (37.50±5.98) (p<0.05) and increasing in HbRbrNROR (66.40±1.12) (p<0.01) (Table 2).

Table 3 shows that the calcium and sodium concentrations were increased in statistically-significant but very small amounts (3–5%) in all experimental groups. Also, potassium levels were slightly further increased (~10% compared to control). Iron and phosphorus concentrations showed a decrease in all experimental groups, to ~60% with the exception of HbRbr where in the case of iron the decrease was less evident.

**Table 3:** Blood ion concentration of control and experimental groups (mean ± SEM).

Parameters	Control	nHb	HbGL	HbBSA	HbRbr	HbRbrNROR	HboATP
Iron (μg/dL)	293.0±22.9	214.8±17.8*#	128.0±10.5*	170.7±7.3*#	232.1±5.8*#	142.6±3.6*	134.3±4.0*
Phosphorus (mg/dL)	21.5± 0.6	16.7±0.7*#	16.8±0.6*	14.2±0.4*#	14.3±0.2*#	13.6±0.5*#	13.6±0.3*#
Sodium (mmol/L)	144.5± 0.2	149.7±0.4*	150.2±0.2*	151.7±0.4*#	151.4±0.3*#	152.6±0.2*#	151.8±0.9*
Potassium (mmol/L)	3.6± 0.0	3.9±0.0*	4.0±0.0*	4.1±0.0*	4.2±0.0*#	4.2±0.0*#	4.2±0.0*#
Calcium (mg/dL)	1.3±0.0	1.3±0.0	1.3±0.0*	1.3±0.0*	1.3±0.0	1.3±0.0*	1.3±0.0*

* Significant at *p* < 0.05 as compared to Control

# Significant at *p* < 0.05 as compared to HbGL

**Fig 1:**
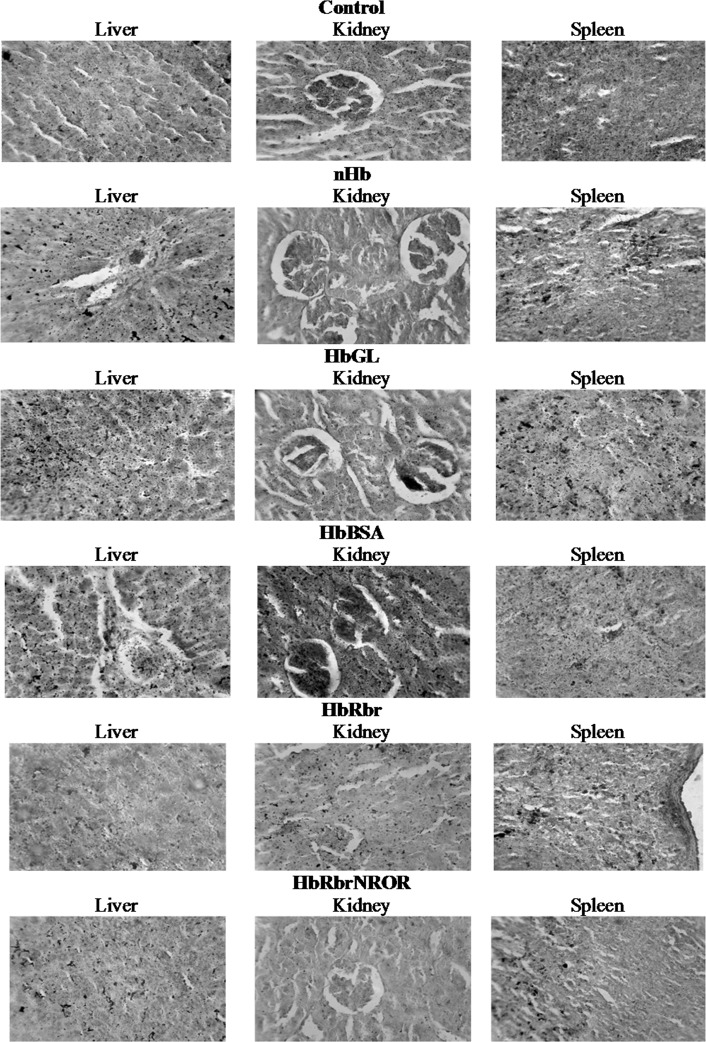
Iron histochemical analyses.

Transferrin concentrations were increased by ~ 40% in all experimental groups, except the nHb (Table 4). Iron histochemistry analysis revealed large iron-phosphorus complexes stored in liver (nHb, HbGL, HbBSA), kidney (HbBSA, HbRbr, HbGL) and spleen (nHb, HbGL, HbBSA) (Fig 1 and Table 5).

**Table 4:** Transferrin, total proteins, and glucose in control and experimental groups. Values are expressed as mean ± SEM

Parameters	Control	nHb	HbGL	HbBSA	HbRbr	HbRbrNROR	HboATP
Transferrin (U/L)	134±2.5	135 ± 2.7#	176 ± 3.3*	167 ± 3.0*	156 ± 4.8*#	184 ± 5.2*	173 ± 1.6*
TP (g/dL)	7.9± 0.3	8.2± 0.1#	23.0±0.3*	23.0±1.0*	24.4±0.4*	24.7±0.4*#	24.2±0.3*#
Glu (mg/dL)	147.8±3.8	148.8±4.0	146.8±2.3	146.7±1.3	148.4±2.2	145.8±2.5	148.3±5.7

* Significant at *p* < 0.05 as compared to Control

# Significant at *p* < 0.05 as compared to HbGL

**Table 5:** Comparison of the iron deposits in liver, spleen and kidney in Control and experimental groups. The structures were scored for their intensities of iron deposition in the range of + (normal aspects of the analyzed structures) and ++++ (the most intensely stained areas). The absence of iron deposits was marked with–(negative).

Experimental groups	Liver	Spleen	Kidney
Control	+	++	+
nHb	++	+	-
HbGL	++++	++++	+
HbBSA	+++	++	++++
HbRbr	-	+	+
HbRbrNROR	+	+	-
HboATP	+	+	-

The total protein content was only slightly increased in the native hemoglobin group (8.23±0.12) (p>0.05), whereas it was ~tripled in all the other groups (Table 4).

Blood glucose concentrations remained at normal levels (Table 4).

Renal function parameters (creatinine, urea, uric acid) (Table 6) remained at normal levels. Uric acid showed slightly lower concentrations in HbRbr (3.63±0.04) (p<0.001) and HboATP (3.61±0.08) (p<0.001) groups, but with no clinical significance.

**Table 6:** Renal function parameters of control and experimental groups. Values are expressed as mean ± SEM.

Parameters	Control	nHb	HbGL	HbBSA	HbRbr	HbRbrNROR	HboATP
Crea (mg/dL)	1.1±0.0	1.1 ±0.0	1.1±0.0	1.1±0.0	1.2±0.0	1.2±0.0#	1.2±0.0
Urea (mg/dL)	37.3±3.0	38.4±1.2#	32.8±1.4	43.4±3.4#	39.8±1.6#	34.5±0.7	34.1±0.4

# Significant at *p* < 0.05 as compared to HbGL

Acid-base equilibria were significantly influenced by all HBOCs. The partial pressure of carbon dioxide was decreased by all molecules, in contrast with the partial pressure of oxygen, which was increased. Taking into consideration the partial pressure of carbon dioxide, the alkaline reserve (cHCO_3_^-^) was proportionally influenced. All these parameters are reflected in blood pH values, which are higher than control animals by very small but statistically significant amounts (Table 7). Furthermore, blood pH values are in a relationship with bases excess (as buffers and from extracellular fluids).

**Table 7:** Acid base equilibrium of control and experimental groups. Values are expressed as mean ± SEM.

Parameters	Control	nHb	HbGL	HbBSA	HbRbr	HbRbrNROR	HboATP
pH	7.3±0.01	7.4±0.01*	7.4±0.01*	7.4±0.01*	7.4±0.01*#	7.4±0.01*	7.4±0.01*#
pCO_2 (mmHg)_	42.9± 1.3	38.5±0.6*	37.8±1.0*	39.5±0.9*	40.9±0.9#	38.8±0.9*	39.7±0.4
pO_2 (mmHg)_	39.3± 1.5	45.8±0.9*	44.7±0.7*	47.1±1.0*#	42.4±1.7	43.6±1.6*	45.3±1.2*
cHCO_3 (mmol/L)_	26.0± 0.3	25.5±0.5	25.8±0.3	26.1±0.5	26.2±0.8	26.0±0.6	26.1±0.4
BE(ecf) (mmol/L)	0.7± 0.2	0.2±0.3#	1.8±0.1*	1.3±0.5	3.5±0.4*#	1.0±0.53 ^a^	2.1±0.5*
BE(b) (mmol/L)	1.2± 0.3	0.9±0.3#	1.8±0.2	1.0±0.3#	2.7±0.5*	2.6±0.2*#	2.7±0.5*

* Significant at *p* < 0.05 as compared to Control

# Significant at *p* < 0.05 as compared to HbGL

Serum catalase activity (CAT) activity was essentially doubled in all groups vs. the control (Table 8); this may, however, involve partial masking by hydrogen peroxide-consuming activity (catalase or peroxidase) displayed by the HBOCs themselves[7, 37, 42]. The thiobarbituric acid reactive substances (TBARS) presented negligible (~1%) increases with nHb, HbGL and HbBSA, and somewhat higher (15–25%) in the rest of the samples.

**Table 8:** Oxidative stress status of control and experimental groups, reflected by catalase and thiobarbituric acid reactive substances. Values are expressed as mean ± SEM

Parametes	Control	nHb	HbGL	HbBSA	HbRbr	HbRbrNROR	HboATP
CAT (U/mL)	118.5± 16.1	279.7±49.2*	289.7±73.7	287.4±95.0	262.7±8.3*	343.9±148.8	238.9±4.3*
TBARS (nmol/mL)	1.74±0.03	1.77±0.01*#	1.79±0.02*	1.80±0.01*#	2.19±0.08*#	2.16±0.05*#	2.00±0.04*#

* Significant at *p* < 0.05 as compared to Control

# Significant at *p* < 0.05 as compared to HbGL

**Fig 2:**
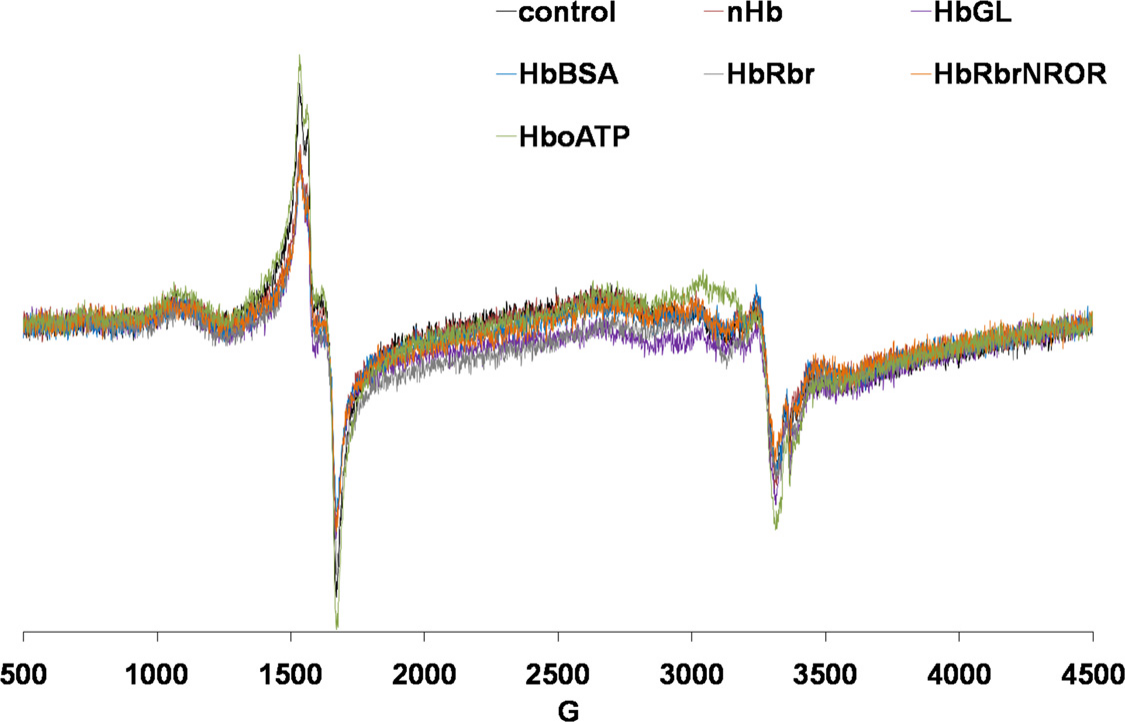
EPR spectra of Wistar rat venous blood (c.f. Materials and Methods) measured at 100K; the *g* values are indicated by arrows.

In the EPR spectra (Table 9, Fig 2) the two key signals were the g~6 corresponding to metHb and the g~2 corresponding to free radicals–both of which are indicative of increases in oxidative stress. Native Hb as well as HbGL induce measurable increases in both these signals. By contrast, HbRbr and HbRbrNROR, previously noted for their *in vitro* ability to scavenge hydrogen peroxide,[39] reduce both EPR signals to the levels seen in the controls. HbBSA provides negligible improvement over HbGL in terms of the metHb signal, and none in terms of the free radical. HboATP perhaps expectedly yields by far the highest free-radical signal of all samples, as well as an increased metHb signal–in line with the previously described increased prooxidant reactivity[5].

**Table 9:** EPR data for blood collected after injection of animals (n = 6) with 200 μL HBOC candidate. Percentage changes relative to the control group are shown, for the concentrations of metHb (g~6), free radical (g~2) and transferrin (g~ 4.1) signals, respectively.

	metHb	S.D.	free radical	S.D.	transferrin	S.D.
Hb	+34.1	17.3	+7.5	1.3	-2.0	3.7
HbGL	+37.6	20.5	+7.3	2.8	-2.0	2.2
HbBSA	+33.1	20.4	+7.9	3.3	-1.4	3.2
HbRbr	-0.1	13.2	-11.1	1.7	-1.6	3.8
HbRbrNROR	+1.9	12.9	-24.5	1.0	+1.0	3.9
HboATP	+21.6	14.7	+34.8	1.4	-1.7	2.3

## Discussion

While most previous *in vivo* studies have been focused on one particular product[28, 29], the present report attempts to examine in parallel a set of products based on crosslinking protocols (mainly glutaraldehyde), differing in their prooxidative parameters.

### Immunohaematology and Coagulometry

Hematological parameters showed significant variations in all experimental groups. However, except those associated with the erythrocytes, the values remained confined within the normal range. RBC counts, HGB and HCT were slightly above normal, while the other red cell parameters were practically unchanged. Erythrocytosis, the increased concentration of RBCs, is commonly described in acute and chronic circumstances. Acute circumstances are associated with dehydration and excitement response. Chronic circumstances, that are unlikely here because of the short time span of experiment, might include chronic hypoxemia, kidney circulatory condition and other conditions associated with intensified erythropoietin synthesis. Hypoxemia is also unlikely since pO_2_ was significantly increased in all experimental groups. In acute circumstances, the excitement response is also associated with leukocytosis dominated by elevated neutrophils and lymphocytes; therefore, in the present circumstances, it is more likely that the slight erythrocytosis is a side effect of a mild dehydration.

WBC blood variation reflects the leucocyte dynamics, the balance between tissue demands and bone marrow synthesis. The leukocyte populations have different functional and diagnostic significance, but they are all part of immune system response; therefore, their variations should be correlated with plasma immunological parameters[43].

All the tested HBOCs induced a systemic inflammatory response, as suggested by the two-fold increase in fibrinogen concentration and almost four-fold increase in CRP. Both acute phase proteins are produced by liver in response to IL 6, synthesized mainly by activated macrophages. Usually, IL 6 is controlled *via* production of IL 1, responsible for fever and increased release and production of granulocytes[43]. However, increased granulocyte concentration was found only in HbRbrNROR, and the value was not above the normal range. Why hemoglobin compounds were unable to elicit a significant granulocytic response associated to systemic inflammatory syndrome, remains unclear.

Blood lymphocytes are key elements in specific immunity; low lymphocyte concentration in blood is usually associated with immune suppression. Notably, despite the fact that the values obtained in the present experiment were lower than those of the control, they were confined within the normal limits. Additionally, in this case, elevated levels of both IgA, IgG, and IgM, that were found invariably in all experimental groups, proved an enhanced humoral-specific immune response. It is widely accepted that the enhancement of specific immunity is not necessarily associated with increased lymphocyte concentration in the blood; therefore, the low values may more likely be related to intense demands than to the reduced production[43]. Importantly, the BSA and Rbr/NROR chemical modifications do not appear to add to the intrinsic immunogenicity of HbGL, while indeed HbGL preparations from other groups have been reported to show no adverse immune reactions in humans or elsewhere[21, 44].

Platelets are not only key factor of hemostasis; reactive thrombocytosis is also a common finding in systemic inflammatory response. Increased platelet production is triggered by intensified thromboplastin synthesis in response to inflammatory cytokines[45, 46]. Clotting parameter values (fibrinogen, PT, aPTT) confirm that blood substitutes may also modulate the immune response *via* platelet activation signaling, which is at the intersection of hemostasis and the immune reaction[46]. However, the variations compared to the healthy control animals do not suggest pathophysiological relevance. Comparatively, a study on controlled hemorrhagic model on pigs also revealed slightly abnormal values for clotting parameters (fibrinogen, PT, aPTT)[27, 28].

### Blood biochemistry

The increases in total protein concentrations correlate with the higher levels of immunoglobulins and transferrin. Introducing a new heme protein in the vascular system is naturally expected to lead to a larger amount of transferrin as a means to scavenge excess iron. The higher calcium concentration in serum may help maintain an optimal binding between the hemoglobin molecule and its membrane receptors. Sugar metabolism is not affected in the experiments described here; glycaemia remained constant, as also noted in other experiments, performed under different conditions[27, 28].

The serum creatinine and urea levels provide no evidence for problems associated with the kidney. In previously-described posthemorrhagic shock transfusion experiments, HbGL demonstrated a mild but a significant serum creatinine level increase, indicative of acute renal dysfunction because the hemorrhagic shock is associated with a low blood kidney perfusion which determines a decrease of the glomerular filtration rate and even tubular necrosis[47].

Taking into consideration the ion composition of the HBOC, which is based on sodium and potassium buffer salts, it is normal that serum levels of these cations are increased. For the rest, the iron metabolism interferes with phosphate, as the two ions generate an iron-phosphate complex in ferritin, which accumulates into the specific tissues, including liver, spleen and kidney (see also the Iron Histochemistry paragraph).

The acid-base equilibrium was slightly perturbed, with trends of metabolic alkalosis depending on the HBOC. An increased partial pressure of oxygen and a decreased partial pressure of carbon dioxide indicate a good interaction between our HBOCs and respiratory gases. Bicarbonate variations are not significant but there was a tendency of increase in HbRbr, HbRbrNROR and HboATP, which may indicate a possible exposure to a metabolic alkalosis. These events are also confirmed by the trends seen in the base excess from extracellular fluids (BE-ecf) and base excess with buffer properties (BE-b). In contrast, some experiments indicate that after a hemorrhagic trauma in rats, HBOC administration provided a significant improvement in oxygen consumption and acid-base equilibrium, but with a decreased base deficit[47] and increased carbon dioxide concentration in blood and tissues. In preclinical studies on dogs, the authors observed the same base deficit, arguing the possibility of an acidosis, attributed to the loss of buffers[29].

### Iron Histochemistry

The histochemical data illustrate different degrees of accumulation of iron complexes depending on the specific HBOC. The higher occurrence of high iron-phosphorus complexes in liver (nHb, HbGL, HbBSA), spleen (nHb, HbGL, HbBSA) and kidney (HbBSA, HbGL) demonstrate different mechanisms of interaction between our blood substitutes and iron metabolism, in agreement with Baek et al. (2012)[48]. Notably, HbRbrNROR and HbRbr induce distinctly lower (and in the normal range) iron deposits than the simpler HbGL, or even than HbBSA. This may be interpreted as evidence that the redox protection offered by Rbr extends as far as preventing acute deposition of iron at the respective organs.

### EPR and Oxidative Stress Evaluation

The CAT and TBARS levels at 48 hours after treatment with HBOC reveal no significant increase in the nHb group–despite the previously-documented *in vitro* prooxidant reactivity. Increased levels of TBARS in serum are generally associated with higher activity levels of serum catalase (CAT)[49], and this is indeed observed in Table 9. By contrast with the TBARS and CAT measurements, the EPR spectra collected on blood samples extracted 10 minutes after administration of the HBOCs to the animals, reveal a picture closely mirroring the previously-reported *in vitro* prooxidant reactivity[39, 50, 51].Thus, nHb and HboATP display distinctly higher EPR signals, while by contrast HbRbrNROR shows no change compared to the controls other than a slight decrease in the free radical signal.

## Conclusions

Reported here is a complex evaluation of a range of physiological parameters in rats exposed to a library of HBOC candidates displaying distinctly varying prooxidative reactivity, with the hypothesis that less prooxidant products will display less negative pathophysiological effects. This library has included native Hb as a well-documented case with measurable negative effects. Glutaraldehyde-crosslinked Hb was taken as a reference point, since similar products were approved for human use in South Africa, and yet prooxidant reactivity is also well documented. HboATP was previously reported to display increased *in vitro* prooxidant reactivity, and was thus expected to fare worse in the *in vivo* tests compared to HbGL. HbBSA, HbRbr and HbRbrNROR were previously described to display lower levels of prooxidant reactivity than HbGL, and it was therefore expected that at least in part they would fare better in the *in vivo* tests, compared to HbGL; the added complexity was, however, that the extra proteins in these preparations (albumin, Rbr, NROR) may interfere with the immune system. The biochemical and physiological parameters monitored in this study for all these HBOCs revealed no pathological variations. The tested HBOCs tend to behave very similarly to each other; in only a few instances, such as paramagnetic signals detected by EPR, or the increases in iron deposits in various organs, did the redox properties appear to lead to distinct differences in measurable biological parameters. Thus, in terms of these two parameters, HbRbrNROR appears to indeed show the expected advantages over HbGL, without notable immunological costs; to some extent this is also true of HbBSA. These data offer support for further hemorrhagic-shock studies with the better-performing candidates.

## Supporting Information

S1 File Supporting Information data. Raw data supporting the findings in the manuscript Tables.
(XLSX)

## Abbreviations

HBOC: hemoglobin based oxygen carrier
Hb: hemoglobin
DBBF: bis(3,5-dibromosalicyl) fumarate
oATP: adenosine triphosphate oxidized by periodate
PEG: polyethylene glycol
DSS: disuccinimidyl suberate
HUVEC: human umbilical vein endothelial cells
Rbr: rubrerythrin
nHb: native hemoglobin
HbGL: hemoglobin polymerized with glutaraldehyde
HbBSA: hemoglobin copolymerized with BSA
HbRbr: hemoglobin copolymerized with rubrerythrin
HbRbrNROR: hemoglobin copolymerized with rubrerythrin and NROR. HboATP—hemoglobin polymerized with oATP
RBC: red blood cell
IDR: Intra Dermic Reaction
